# Perspectives in Pediatric Ambulatory Anesthesia: Part 2—One Center's 15 Year Experience Improving Quality and Safety Outcomes

**DOI:** 10.1002/pan.70252

**Published:** 2026-07-03

**Authors:** Jennifer L. Chiem, Elizabeth E. Hansen, Kayla Reece, Samuel M. Vanderhoek, Sanjay R. Parikh, Paul A. Merguerian, Lynn D. Martin

**Affiliations:** ^1^ Department of Anesthesiology & Pain Medicine Seattle Children's/University of Washington School of Medicine Seattle Washington USA; ^2^ Department of Perioperative Services Seattle Children's Seattle Washington USA; ^3^ Division of Pediatric Anesthesiology, Department of Anesthesiology and Critical Care Medicine Johns Hopkins University School of Medicine Baltimore Maryland USA; ^4^ Department of Otolaryngology‐Head and Neck Surgery Seattle Children's/University of Washington School of Medicine Seattle Washington USA; ^5^ Department of Urology Seattle Children's/University of Washington School of Medicine Seattle Washington USA

**Keywords:** ambulatory surgery, enhanced recovery, opioids, pediatric anesthesia, positive deviance, quality improvement, sustainability

## Abstract

**Introduction:**

Pediatric ambulatory surgery has become the dominant model of surgical care in the United States, driven primarily by economic forces. There is variability in regional practice patterns, quality improvement cycles, and outcomes. Opportunity exists to overcome knowledge gaps and provide sustainable pathways of quality improvement. Our unique capability of describing the evolution of our pediatric ambulatory quality improvement practice allows us to contribute a single center's perspective.

**Methods:**

We chose to complete a comprehensive retrospective review of our quality improvement process, outcome, and balancing metrics contained in our electronic health record (EHR) from our free‐standing pediatric ambulatory surgery center (ASC) from July 2010 through December 2024. A commercial software system extracted de‐identified, aggregated health data from the system's EHR. The data are processed and presented in statistical process control charts. This methodology allows clinicians to distinguish between common cause and special cause variation.

**Results:**

Improvement themes (opioid‐free anesthesia and stewardship, enhanced recovery, environmental efforts, positive deviance, and learning healthcare system) are described. Improvements in all six domains of quality (effectiveness, efficiency/timeliness, patient experience, equity, and safety) are illustrated with reliable sustainability. Our system achieved approximately a 13‐fold increase in quality improvement (QI) project completion rate with self‐serve, real‐world data access; enabling the team to take on improvement tasks previously deemed too big, lengthy, or risky to complete.

**Discussion:**

We provide preliminary evidence that these methods may be generalizable. Requirements include engaged leadership, a standard framework for improvement with experienced leadership or accessible support, and easy access to real‐world electronic medical record data (i.e., learning healthcare system [LHS]). Lastly, leaders must create a culture supportive of teamwork, change, and continuous improvement. Systems facilitate adoption and hinder resistance to standards, always with implementation and sustainability in mind. Meaningful, large‐scale improvements in healthcare outcomes require collaboration across LHSs.

**Abbreviations**
ASA: American Society of Anesthesiology
ASC: ambulatory surgery center
EHR: electronic health record
ERP: enhanced recovery program
GHG: greenhouse gas
LHS: learning healthcare system
MME: morphine milliequivalent
N_2_O: nitrous oxide
OFA: opioid‐free anesthesia
OR: operating room
OSA: obstructive sleep apnea
PACU: post anesthesia care unit
PD: positive deviance
PDSA: Plan‐Do‐Study‐Act
PONV: postoperative nausea and vomiting
QI: quality improvement
SCV: special cause variation
SPC: statistical process control
T&A: adenotonsillectomy
US: United States

## 1 Introduction

Pediatric ambulatory surgery has become the dominant model of surgical care in the United States (US), driven by economic forces (insurance mandates, reimbursement, and cost savings). There is significant variability in these practices and disconnects remain between research (i.e., published literature) and the setting where most pediatric perioperative care is delivered [1, 2]. Vanderhoek et al. [1] identified important and growing gaps in our knowledge that must be closed.

Healthcare quality improvement (QI) has itself undergone significant evolution over the last half century. The true founding moment for contemporary healthcare QI occurred in October 1965 [3]. Avedis Donabenian was commissioned to review the research on quality assessment and his report, published in 1966, defined the ‘structure, process, and outcomes’ in healthcare quality [4]. Gaining traction in the 1970–1980s, many methodologies for healthcare QI, including Lean, Six Sigma, and Model for Improvement, were adapted from manufacturing for healthcare [5, 6, 7]. A systemic review highlighted the lack of methodological rigor and reported effects from healthcare QI publications [8]. Only 60% of QI publications sufficiently documented their Plan‐Do‐Study‐Act (PDSA) for inclusion, only 27% described a specific, quantitative aim, and only 4% adhered to all four PDSA methodological features [8]. Developed in 2006 by the Institute of Medicine, the concept of a Learning Healthcare System (LHS) applies “the best evidence for the collaborative healthcare choices of each patient and provider and to ensure innovation, quality, safety, and value in healthcare.” [9] The LHS structure aligns science, informatics, incentives, and culture to drive continuous improvement and innovation. A systemic review demonstrated researchers' interest in the LHS, but there has been little focus on the impacts of this new QI paradigm [10].

With the rapid growth in ambulatory perioperative care, there has been a significant investment in ambulatory QI. Quality indicators for ambulatory surgery have been described in three categories: (1) patient reported outcome measures, (2) efficiency measures (room utilization, case length accuracy, etc.), and (3) value‐based measures (e.g., safe, efficient, effective care at lower costs) [11]. Pain and its management and perioperative emergencies are common quality indicators [12, 13]. Recent assessments of medical malpractice insurance closed claims suggest higher than expected patient and procedural complexity in ambulatory surgery centers (ASCs) [14].

Clinicians and researchers have made similar efforts to define quality in pediatric anesthesia [15]. Historically, the primary areas of focus have been on safety and pain management [16], although Olbrecht et al. [17] advocated for expansion and the need for standardized definitions, benchmarks, and outcomes. They noted that most published pediatric anesthesia QI work originates in academic centers, while much of the care is delivered in the community, and advocated for the creation of a pediatric anesthesia quality verification program [18]. While meritorious, their proposal fails to recognize the mandatory partnership in the perioperative environment between surgeons, anesthesiologists, anesthetists, and perioperative nurses.

### 1.1 Specific Aim

In our series of perspectives articles, we completed a comprehensive retrospective review of our process, outcome, and balancing metrics contained in our electronic health record (EHR) from our pediatric ASC since its opening in July 2010 through December 2024. In part 1 we focus our observations on eligibility criteria, volume, acuity, and outcomes [2]. In part 2, we review and summarize our multiple, extensive, and ongoing QI initiatives and their outcomes.

## 2 Methods

A more detailed review of our methods was described in part 1 of this series [2]. We briefly summarize here.

### 2.1 Context

Seattle Children's is a pediatric health system affiliated with the University of Washington with 345 licensed beds, 24 ORs, and 4 procedural suites. This institution utilizes Lean principles; standardized practice, waste minimization, visual tracking of information, and continuous iterative improvement using PDSA cycles to enhance healthcare outcomes. The ASC affiliated was launched to serve as a site where clinicians and nursing staff could develop standardized protocols derived from evidence‐based literature to enhance clinical outcomes. Multimodal analgesia has been a foundation of this practice supplemented by regional anesthesia whenever possible. Protocol compliance is facilitated by customized, case‐specific templates built into the EHR. Clinical and improvement teams gain real‐time access to data via a software solution (AdaptX Seattle, WA, USA) that aggregates deidentified data from the EHR and applies statistical process control (SPC) analytical methods to distinguish between random variation (noise) and special cause variation (SCV) [19, 20]. This ability to create custom cohorts and visualize key process and outcome metrics promotes rapid understanding and increased rates of improvements [21]. The healthy patient population, low acuity cases with high volumes, standardized clinical practices, and accessible process and outcomes data all facilitate the continuous improvement in processes and outcomes.

### 2.2 Measures

Several previously described SPC chart types were used in this review [2]. The P‐chart was used for the incidence of opioid‐free anesthesia (OFA), enhanced recovery program (ERP) measures, and PACU normothermia (defined as initial temperature > 36°C). A C‐chart was used for surgical case counts. An X‐bar chart was utilized for PACU maximum pain score, surgical preparation time (defined as time from anesthesia ready to incision), CO_2_e (defined as CO_2_e kg/min for every anesthetic), morphine milliequivalent (MME defined as the total amount of opioids received in morphine milligram equivalents) both intraoperatively and in the PACU, and prescribed postoperative opioid dose counts. Each X‐bar chart has a paired S‐chart that displays the standard deviation of this data. A U‐chart (a variant of a C‐chart displays nonconformities per unit‐cases), was used for postoperative nausea vomiting (PONV) rate (defined as antiemetic administration in the PACU) and inbound postoperative family phone calls. A Funnel chart (an X‐bar, S, P, or U chart sequenced by order of subgroup from smallest to largest with control limits) was used for surgery preparation time and 30‐day re‐admission rate for adenotonsillectomy (T&A) by race. Prime corrections, used for data with over‐dispersion [20], were utilized for OFA, ERP measures (pre‐operative acetaminophen and carbohydrate drink), and PACU normothermia rate.

### 2.3 Ethical Consideration

The series of QI projects and this specific summary were submitted to Seattle Children's Institutional Review Board. The board considered this QI work. No further action was required.

## 3 Results

### 3.1 Historical Review of Quality ASC Care Improvements

Since opening, the Bellevue ASC has been a leader in innovation via continuous QI for the organization. The list of QI milestones and events and their associated timeline are found in Table 1 and Figure S1. The anesthesia team prioritized the use of multimodal analgesia and extensive use of regional anesthesia to foster opioid‐sparing anesthesia protocols. All ASC staff demonstrated their commitment to developing, adhering to, and improving processes to deliver better outcomes. Throughout the evolution of this practice, several improvement themes emerged.

**TABLE 1:** Timeline and description of major quality improvement milestones and external events.

Annotation	Date	Details
QE 1	7/20/2010	ASC opens; patient flow checklists launch
QE 2	7/31/2010	Morning Team Huddle launch
QE 3	10/31/2011	Safety checklists (WHO Timeout modification) launch
QE 4	4/2/2012	T&A Standard #1
QE 5	9/1/2014	T&A Standard #2
**4/1/2015**		**EHR #1 Launch**
**5/3/2015**		**ASC OR closure for renovations**
**6/29/2015**		**ASC ORs re‐opens**
QE 6	1/1/2016	Desflurane stopped; AdaptX data launch
QE 7	5/1/2016	Hypothermia project
QE 8	8/1/2016	Anesthesia Leader Daily Standard Work
QE 9	10/3/2016	T&A Standard #3
QE 10	12/1/2017	Inguinal hernia repair project
QE 11	6/18/2018	T&A trials launch
QE 12	10/1/2018	Top 12 procedure OFA replication start
QE 13	1/1/2019	ASC‐wide OFA; GHG socialization
**4/1/2019**		**Planned hospital admit with transfer policy approved**
QE 14	4/1/2019	GHG early adopters join team
**5/18/2019**		**Partial hospital OR closure for renovations**
**2/25/2020**		**Hospital ORs re‐open**
**6/30/2019**		**T&A + ASC OFA manuscripts—Franz et al. x 2**
QE 15&16	10/1/2019	Oto T&A lidocaine project + GHG departmental education session
QE 17	11/1/2019	Anesthesia machine FGF default decreased
**3/13/2020**		**ASC closure for COVID**
**5/14/2020**		**ASC Re‐opens with COVID case restrictions and processes**
QE 18	5/14/2020	Morning (virtual) all team safety huddle launch
QE 19	7/1/2020	Oto T&A equity PDSA
QE 20	9/1/2020	Urology chief surgery preparation process trial
QE 21	10/1/2020	ERP department educational sessions
**10/4/2020**		**EHR #2 launch**
QE 22	1/1/2021	Oto T&A opioid stewardship project
QE 23	1/25/2021	ERP phase 1; Urology (prep) & Oto (postop opioids) div. meetings
QE 24	3/1/2021	T&A Ketamine 0.2 mg/kg trial
QE 25	4/1/2021	ERP phase 2 & GHG decision support tool launch
QE 26	6/1/2021	Oto post‐op opioid standard orders #1
QE 27	7/19/2021	ERP phase 3 launch
QE 28	9/6/2021	T&A—increase Ketamine 0.4 mg/kg
**10/31/2021**		**Urology surgery preparation data stop – Fernandez et al.**
QE 29	12/1/2021	Project SPRUCE launch
**12/31/2021**		**ERP data ends—Martin**
QE 30	1/1/2022	Ketamine replication other procedures; Oto postop opioids std. #2
QE 31	1/4/2022	Unplugged nitrous from all machines
**3/1/2022**		**COVID restrictions end**
**6/302022**		**Oto postop data ends LHS ref**
QE 32	7/16/2022	ERP phase 4—PACU RN Dexmedetomidine approval
**9/30/2022**		**OFA follow up data ends—Martin et al.**
**10/31/2022**		**GHG data collection stop—Hansen et al.**
QE 33	12/1/2022	Oto post‐op opioid standard orders #3
**1/1/2023**		**Planned Admit Policy ends**
QE 34	4/1/2023	Urology ERAS project
QE 35	5/1/2023	T&A‐Increase Ketamine 0.5 mg/kg
**6/30/2024**		Positive deviance data ends—Low et al.

*Note:* Bold indicates major events.

#### 3.1.1 Improvement Themes

##### 3.1.1.1 Opioid Free Anesthesia and Stewardship

After > 7 years of opioid sparing practice, it was logic to take the final step to OFA. The team, challenged to add anesthesia value, opted to replace high‐cost intravenous acetaminophen with lower cost dexmedetomidine in our T&A protocol. After several iterative PDSA improvement cycles and partnering with our Otolaryngologists to add ketorolac, an OFA protocol was developed that maintained PACU pain scores and duration while reducing PACU opioid administration and PONV treatment [22]. Subsequent iterative PDSA cycles refined the protocol with the goal to minimize PACU opioid administration [19]. This was accomplished with the introduction and escalation of ketamine dose, yielding PACU opioid rates below the target of 10%. Our limited balance measures (postoperative inbound phone calls, 7‐day return to emergency room, or 30‐day hospital admission) were unchanged. In the editorial accompanying this publication, McDonnell highlighted the methodological rigor of this large‐scale continuous QI work to ask and answer questions using real‐world data [23]. He concludes with “….these findings represent a significant step forward for patient care and, maybe, a guide to bridging the gap between continuous QI and ‘research’.” These same methods were subsequently applied to strabismus and inguinal hernia repair [24, 25].

The team embraced the theoretical (patient and provider opioid use disorder) and real (less PONV) advantages of an OFA protocol. They chose to spread this practice in a phased manner to all remaining ambulatory procedures [26]. After gaining additional experience and confidence, clinicians recognized the OFA advantage for high‐risk T&A patients (i.e., sleep disordered breathing or obstructive sleep apnea [OSA]). Following good OFA outcomes there, the Otolaryngology leadership was granted approval to increase the acceptable ambulatory OSA apnea hypoxia index exclusion threshold from 10 to 15. This change resulted in more than 50 avoided hospitalizations annually [27].

Our surgical colleagues also focused efforts to improve postoperative care, including opioid stewardship, reporting a 43% reduction in opioid dose counts after 2 PDSA cycles without change in balance measures [28]. They subsequently achieved an additional 20% reduction in opioid prescriptions since this publication.

##### 3.1.1.2 Enhanced Recovery Program

Programs designed to specifically improve recovery following surgery, called enhanced recovery after surgery, initially focused on adult patients undergoing complex inpatient procedures, but subsequently expanded to adult ambulatory patients [29, 30]. Pediatric surgeons took notice and embraced these practices [31]. Realizing that many of the key ERP principles (*Pre‐operative*: screening and family education; *Intraoperative*: short‐acting anesthetics, opioid sparing; *Post‐operative*: opioid‐sparing, early feeding) were already integrated into our practice, we launched a plan to improve existing ERP elements and launch new ones. The team was able to achieve 17/19 ERP elements (enhancing 5 existing and implementing 5 new ones) [32]. These efforts resulted in reduced PACU duration and opioid use without change in pain scores and represent the first report of pediatric ambulatory ERP. This program was uniquely applied center‐wide to all patients. Our urology colleagues applied these practices to their patients and achieved 99% OFA, reduced postoperative opioid prescriptions by 50%, and dose counts by 20% without change in any balance measure and sustained satisfaction scores (9.8/10) [33].

##### 3.1.1.3 Environmental Efforts

Healthcare is a significant contributor to pollution and inhaled anesthetic greenhouse gas (GHG) emissions. The United States has the highest per capita healthcare emissions in the world, responsible for 25% of global healthcare emissions [34]. Inspired and led by a clinical champion, our team used a combination of educational initiatives, practice constraints, protocol changes, and access to real‐world data to reach an 87% reduction of GHG from inhaled anesthetics over 5 years [35]. The GHG emissions at the ASC dropped from 0.424 to 0.051 monthly average CO_2_e kg/min.

##### 3.1.1.4 Positive Deviance Methodology

An approach that looks for success rather than errors, failure, and harm, positive deviance (PD) focuses on exceptional performance by individuals or teams and disseminates these practices throughout the system [36]. Real‐time, democratized access to data makes identification of PD easy and facilitates and sustains improvements. Our recent multicenter report demonstrated the potential of this methodology to align with clinicians' desire to provide the best care possible, thereby improving staff engagement [37].

##### 3.1.1.5 Learning Health System

Developed by the Institute of Medicine, the LHS structure aligns science, informatics, incentives, and culture to drive continuous improvement and innovation [9]. Although the concept has generated interest, the impact of this paradigm on outcomes have been limited [10]. The Seattle ASC team realized they were an active LHS and chose to report their findings and positive impacts on clinical system outcomes [38, 39]. With this as a background, examples of ASC data in all National Academy of Medicine quality domains are reviewed.

### 3.2 Ongoing Quality Improvements

#### 3.2.1 Effectiveness

**FIGURE 1:**
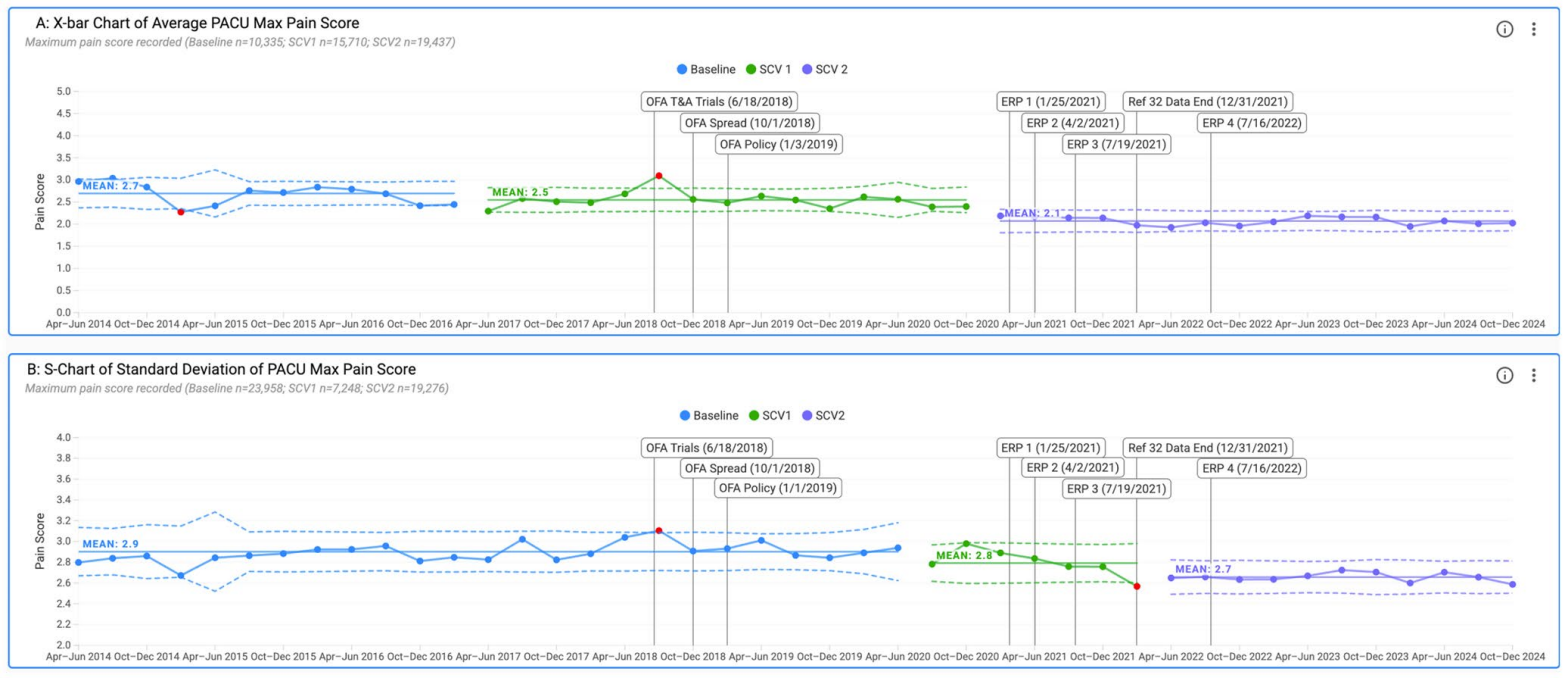
(A) X‐bar and (B) S‐Chart of the quarterly average PACU maximum recorded pain score. Annotations highlight significant QI projects that impacted this metric. Cohorts are separated only when SCV is seen. Red dots represent single point SCV.

Throughout all our QI efforts, our *primary outcome* has consistently been achieving and maintaining acceptable low PACU pain scores. This center‐wide metric seen in Figure 1 (A: X‐bar, B: S‐chart) became available with the launch of our EHR. Improvements in pain scores and reductions (SCVs) in variability were seen. Annotations for the major QI themes (OFA and ERP events) are defined in Table 1.

**FIGURE 2:**
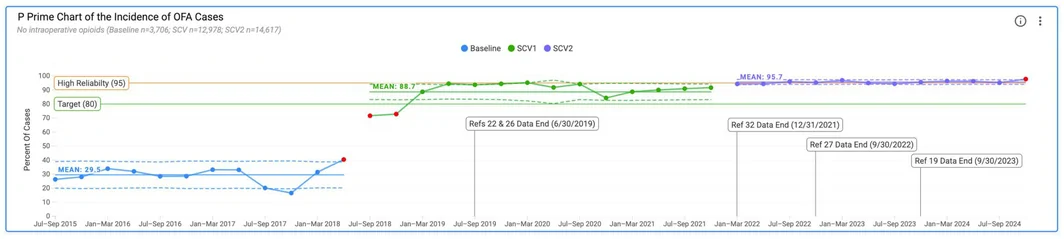
P′‐chart of the quarterly incidence of opioid‐free anesthesia in the ASC. Annotations highlight significant QI projects that impacted this metric. Cohorts are separated only when SCV is seen. Red dots represent single point SCV. The teams original target was 80% and has now exceeded 95%, representing highly reliability.

Opioid related metrics served as *secondary outcome* measures, principal among these was the OFA rate presented in Figure 2. Significant increases were observed with the introduction of OFA trials and again following the ERP projects. *Process metrics* for this work were MME intraoperatively and in the PACU. SCVs were seen in both intraoperative X‐bar (Figure S2A) and S‐charts (Figure S2B). In the PACU, SCVs were seen in both X‐bar (Figure S2C) and S‐charts (Figure S2D). *ERP process measures* were pre‐operative carbohydrate drink and acetaminophen. This process included at‐home administration by parents following instructions from the ASC. These metrics were launched in 1/2021 for pre‐op carbohydrate drink and 9/2021 for pre‐op acetaminophen. SCVs were seen following introduction of pre‐op carbohydrate drink and again in 1/2024 (Figure S3A). No SCV was seen after introduction of the pre‐op acetaminophen (Figure S3B).

Another *secondary outcome measure* was PONV. Based on consensus guidelines for the treatment of postoperative nausea and vomiting [40], the ASC team implemented dual antiemetic prophylaxis (ondansetron and dexamethasone) for defined high‐risk patients and procedures. Guideline compliance for three procedures (T&A, strabismus, and arthroscopy shown in Figure S4A) was high (> 90%), although more variability was evident for strabismus. The rate of early PONV (i.e., treatment with antiemetics in the PACU) is in Figure S4B. There are SCVs for T&A associated with the OFA trials and arthroscopy 1 year prior to the OFA trials. No SCV was seen for strabismus and current rates for all three are very low (< 50 patients/1000 cases).

#### 3.2.2 Efficiency/Timeliness

Operational efficiency is foundational to all ASCs. We designed and built a facility and care model explicitly to increase operational efficiency [41]. This design included the introduction of dual induction rooms for each OR and private PACU rooms; thereby allowing the induction of and emergence from anesthesia to occur outside of the OR. This design necessitated transporting anesthetized patients. To promote safe and efficient patient movement, aviation style ‘challenge‐response’ format patient flow checklists were implemented with the ASC opening [42]. These checklists have been continuously updated by the team driven by changes in clinical practice (EHR introduction) and public health events (COVID‐19 pandemic). Current flow checklists are in Table S1.

**FIGURE 3:**
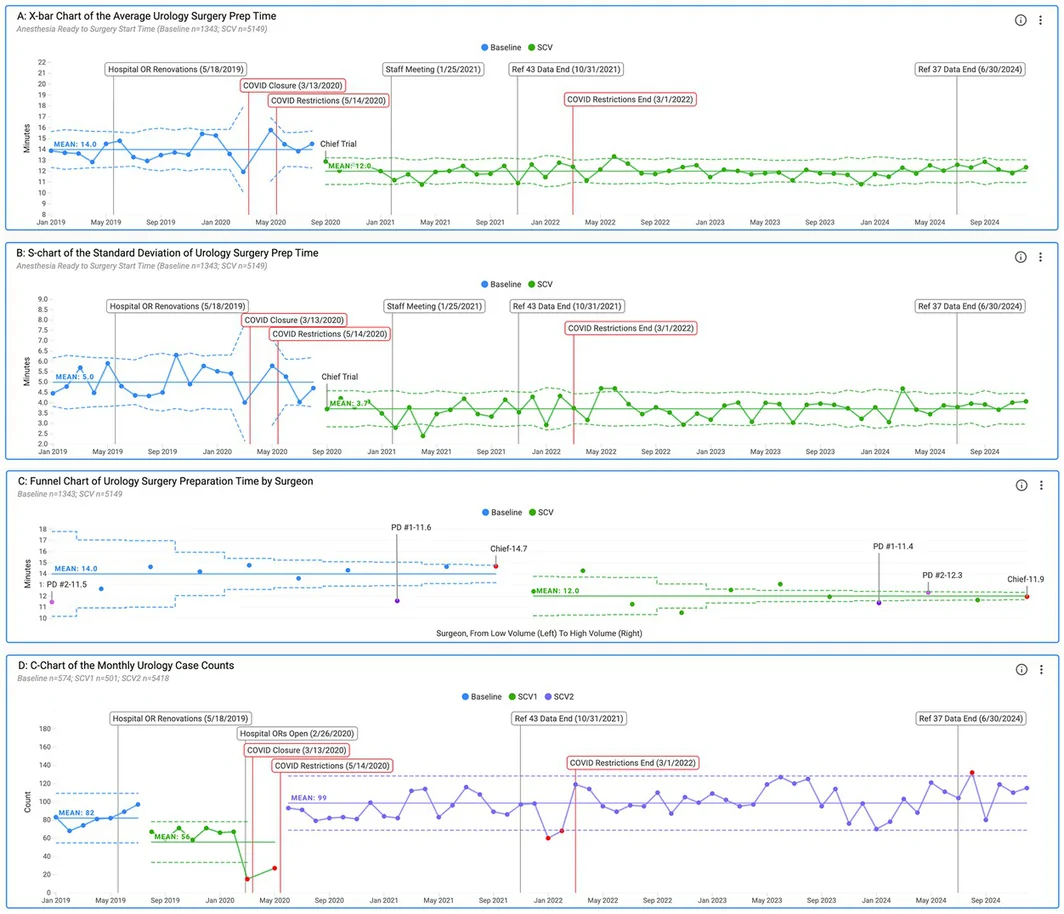
The average Urology surgery preparation time (defined as the time from anesthesia ready until surgery start). Annotations highlight significant QI projects that impacted this metric. Cohorts are separated only when SCV is seen. (A) X‐bar chart, (B) S‐chart, (C) Funnel by urology attending physician (chief and positive deviants are noted by color), and (D) Urology monthly case counts. The gains in monthly volumes have been sustained for over 4 years, including the COVID pandemic.

A specific efficiency improvement project previously reported by our Urology team was stimulated by growing surgical demand adversely impacted by the COVID‐19 pandemic [43]. The authors used PD to find and disseminate best surgical preparation processes (the time between anesthesia ready and incision) first in a trial by the division chief, then disseminated to the entire division. The average urology division surgery preparation time (Figure 3A) and its variability (Figure 3B) decreased during the chief trial and were sustained after dissemination to the division. Figure 3C shows individual surgeon performance in preparation time for the chief (red), positive deviants (purple), and other staff before (blue) and after (green) the chief trial. They achieved their primary outcome, increasing Urology volumes by approximately 20 cases/month (20%) from baseline (Figure 3D).

#### 3.2.3 Patient Experience

Patient experience, originally described as patient centeredness, is defined as providing care that is respectful of and responsive to individual patient/family preferences, needs, and values; ensuring that patient values guide clinical decisions. The opioid crisis in the US has impacted nearly all communities and families either directly or indirectly. In response to these concerns, our surgical colleagues implemented opioid stewardship programs [28, 33]. Opioid prescription dose counts for all ASC patients are shown in Figure S5A,B. The average dose count dropped following the Otolaryngology improvement project and again after the Urology improvements. Only T&A opioid doses are shown in Figure S6A,B. Improvements were made with revision to the postoperative electronic order‐set after each PDSA. Reductions in dose counts were seen with each PDSA cycle and again following retirement of a senior faculty member (Figure S6A). An increase was subsequently seen with recruitment of a new faculty member. A significant reduction in dose count variability is seen after faculty retirement in Figure S6B.

#### 3.2.4 Equity

**FIGURE 4:**
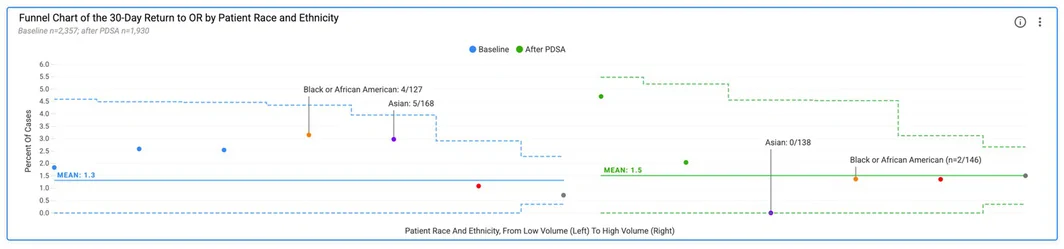
Funnel Chart of 30‐day all cause hospital readmission by patient race and ethnicity for ASC patients. Black/African American and Asian patients had higher readmission rates at baseline and returned to the mean or better after the QI PDSA cycle.

Equity is clinical care delivery that does not vary in quality because of personal characteristics (race, ethnicity, language, geographic location, or socioeconomic status). Observed inequities in care delivery have been uncommon in our standardized practice. One instance of inequity addressed was 30‐day all cause hospital readmission following T&As [38, 39]. The funnel chart of 30‐day hospital readmission rate filtered by race/ethnicity shows higher rates for Black/African American and Asian patients that were resolved after enhancement in the standardized postoperative education (Figure 4). This updated education included a visual hydration chart indicating the number of cups needed to be consumed daily, presumably improving postoperative hydration.

#### 3.2.5 Safety

Patient safety has been the primary concern of anesthesiologists for many decades. Safety in anesthesia after improvements in equipment, medications, and practice processes has reached such levels that mortality is rare and no longer considered a valid safety measure [17]. Morbidity associated with anesthesia and surgery is still present and requires constant vigilance. An example of this vigilance is the use of surgical timeout checklists before starting any procedure. As previously described, our unique ASC facility design and care model necessitated implementation of modified safety checklists [44]. These checklists are broken into 3 separate phases: the ‘Sign‐In’, ‘Time‐Out’, and ‘Sign‐Out’ (Table S2). These modifications encourage parent participation, while challenge‐response formats require active staff engagement, thereby ensuring safer practices.

Another safety intervention implemented with the ASC's opening was the morning all‐team huddle. All team members (surgeons, anesthesiologists, nurses, and technicians) gathered at the start of the day to quickly (< 5 min) review the day's surgery list, calling out any incomplete and unusual processes. This daily in‐person gathering was modified to a virtual format with COVID‐19. The major themes and contents for this huddle are in Table S3.

**FIGURE 5:**
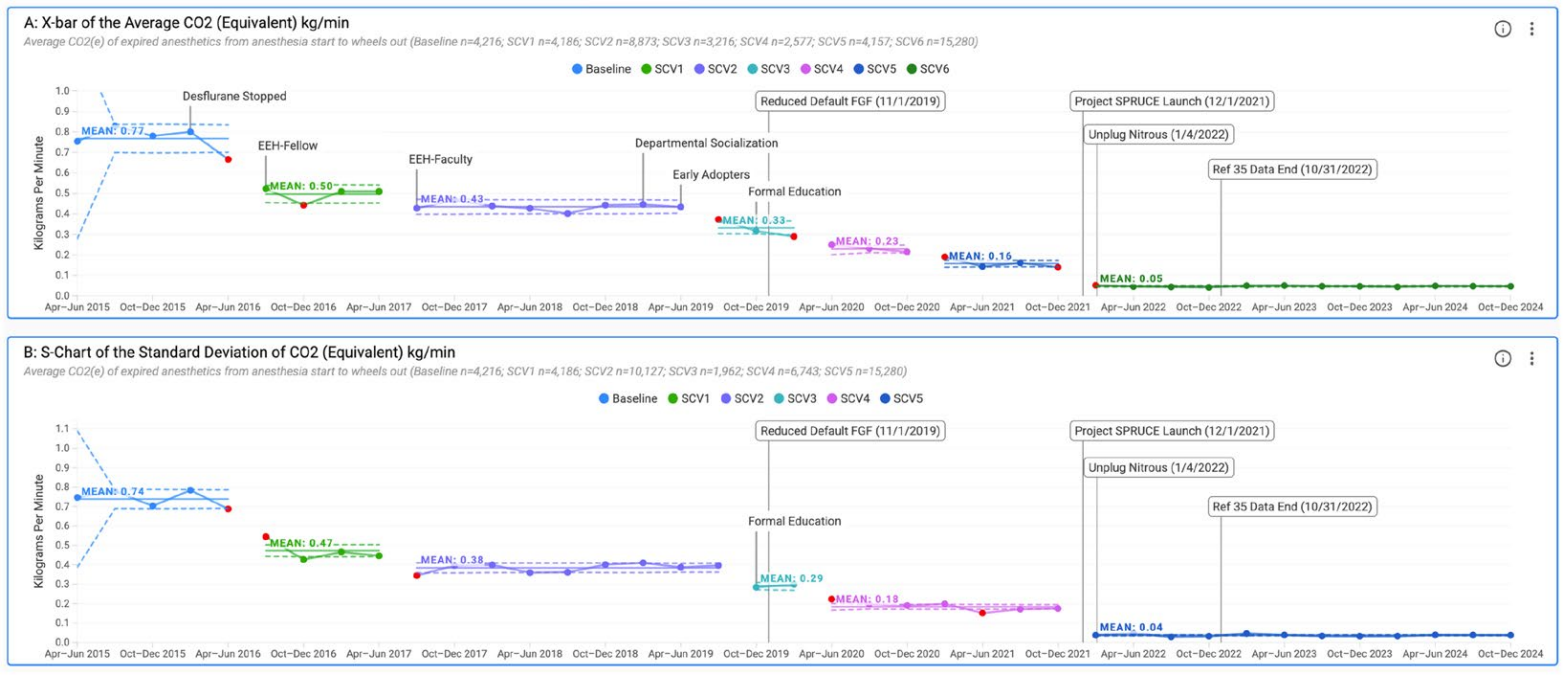
The quarterly average CO_2_e kg/min for the ASC. Annotations highlight significant QI projects that impacted this metric. Cohorts are separated only when SCV is seen. Red dots represent single point SCV. (A) X‐bar chart and (B) S‐chart show significant and sustained reductions in CO_2_e and variability.

Reduction in GHG emissions has been a major safety focus in our institution [35]. Figure 5A displays our continued efforts to reduce average CO_2_e kg/case minute and the variability in practice (Figure 5B). Multiple SCVs are associated with desflurane discontinuation, staff education, EHR alerts, default fresh gas flow reductions, data democratization (Project SPRUCE), and unplugging nitrous oxide (N_2_O) supply. Similar reductions in variability of practice are seen. A major component of these efforts was the education and training of anesthesia providers to first reduce via lower fresh gas flows and ultimately stop use of N_2_O altogether (Figure S7A–C). While there was a small but significant reduction with education, larger SCVs were seen with decision support implementation, data access, and N_2_O supply unplugging.

The final safety (*balance*) measures, included in regulatory reporting, are postoperative complications. Our 30‐day all cause hospital readmission rate, reviewed in part 1 of this series, had SCV associated with approval of planned admissions to mitigate hospital ORs closures for renovations [2]. All other complications, including same‐day and 30‐day return to OR, and 7‐day return to emergency room, didn't show SCV (data not shown). With the implementation of EHR #2 we have been able to monitor inbound postoperative family phone calls to clinics (Figure S8). An SCV drop was associated with the ending of pandemic‐related case restrictions. The incidence of PACU normothermia (a routine safety process measure) in Figure S9 shows an SCV increase associated with a hypothermia prevention project. High compliance (> 90%) has been maintained and reinforced via ERP efforts.

#### 3.2.6 Sustainability

While reports of QI initiatives are frequently published, the sustainability of these improvements is rarely addressed. The estimated failure rate for sustainable improvements is about 70% [45]. The National Health Service in the United Kingdom has suggested adding sustainability as an additional quality domain [46]. Moon et al. [47] have identified factors and mechanisms to sustain hospital‐wide safety initiatives and provided a conceptual framework to assist sustainability. Our systems and leaders have built a culture that reliably, but not universally, delivers sustained improvements. Examples of sustained improvement include OFA (Figure 2), Urology surgery preparation time (Figure 3A,B), GHG reductions (Figure 5A–C), ERP process measures (Figure S3A,B), and PONV (Figure S4A,B). Small reductions in sustainability were evident with PONV‐strabismus only (Figure S4A,B) and postoperative T&A opioid dose counts (Figure S6A,B).

## 4 Discussion

### 4.1 Summary of Key Findings

A summary of our key findings from our review of this center's experience includes: (1) continuous improvement in all six domains of quality healthcare, (2) these improvements are reliably sustained over time, and (3) self‐serve data access accelerated the rate of improvement.

Prior to self‐serve access to EHR data, our motivated team completed 5 improvement events over the first 66 months of ASC operations, commonly using manual data collection and chart reviews, representing 1 event/13.2 months. Following implementation of our electronic data extraction, the same team completed on average 1 event/month, representing approximately a 13‐fold increase. This acceleration enabled the team to take on improvement tasks previously deemed too big, lengthy, or risky to complete.

We have recently reported on the use of positive deviance QI methods to improve quality in two unique pediatric centers [37, 43]. Our GHG reduction efforts have been shared in a QI consortium to reduce GHG emissions in the 10 active pediatric centers, yielding a reduction of > 1 million kg CO_2_e [48]. Finally, a new multicenter QI collaborative focused on improving T&A outcomes has been launched, with all three centers showing early improvements in their first 4 months working together (data not shown). These findings suggest that these practices may be generalizable.

Readers may wonder if these efforts and outcomes are only possible in well resourced, academic centers. Requirements include engaged leadership, a standard framework for improvement with QI experienced leadership or accessible support, and easy access to real‐world EHR data. While the latter is generally associated with financial costs, there is commonly sufficient return via improvements in efficiency and quality to offset these expenses. Leaders must create a culture supportive of change and continuous improvement. Tapping into the intrinsic desire of healthcare professionals to provide excellent service, frontline staff are engaged to work smarter and not harder. Iterative PDSA cycles test ideas and data are used to monitor their outcomes. Systems within the EHR (i.e., templates) are used to facilitate adoption and hinder resistance to standards. Sustainability requires that leaders monitor adherence to the standards with data. Following these practices, the leadership creates a culture that embraces teamwork, continuously strives to do better, and recognizes that change is ‘how we do better’.

### 4.2 Strengths

The use of easily accessible, continuously updated real‐world data in a real‐time manner allowed the teams to rapidly complete multiple PDSA improvement cycles [2, 19]. We use SPC as our primary analytical tool as it provides a visual display of changes over time, allowing prompt local decision making [20, 21]. These improvement efforts occur in a stable and sustained learning culture [10, 38, 39].

### 4.3 Weaknesses

These QI initiatives have several limitations highlighted in part 1 [2], including restriction to a single pediatric center experience which may not reflect the community setting practice where most ambulatory surgeries occur. Furthermore, our practice is exclusively limited to pediatric and young adult patients, and all staff are pediatric‐trained. As previously stated, there are many unique center‐specific facility features that influenced our care model.

We did not perform any supplementary statistical analyses to adjust for confounders, generate effect estimates, and allow for determination of impact assessment [49]. For these reasons, we are not making inferences about populations outside our facility. The pitfalls of prospective, observational before and after QI study designs have been previously described [50]. Easy and transparent access to data, along with a staff mindset that embraces continuous change, is difficult to create and sustain. Our focus on continuous improvement and a learning culture at this ASC is relatively unique [10, 38, 39].

## 5 Conclusions

Over 14 years, our team has made continuous improvements in all six domains of quality healthcare, sustained them over time, and shown that self‐serve data access accelerates the rate of improvement. Early work suggests that these methods may be generalizable beyond our single center. To make meaningful, large‐scale improvements in healthcare outcomes, collaboration across LHSs is needed.

## Author Contributions

Jennifer L. Chiem and Kayla Reece: This author helped with the acquisition and interpretation of data, participated in the drafting and revision of the content, approved the final version, and agrees to be accountable for all aspects of the work. Elizabeth E. Hansen, Sanjay R. Parikh, Paul A. Merguerian, Lynn D. Martin: This author helped with the project design, acquisition and interpretation of data, participated in the drafting and revision of the content, approved the final version, and agrees to be accountable for all aspects of the work. Samuel M. Vanderhoek: This author helped with the project design, interpretation of data, participated in the drafting and revision of the content, approved the final version, and agrees to be accountable for all aspects of the work.

## Funding

The authors have nothing to report.

## Ethics Statement

The Institutional Review Board from Seattle Children's reviewed and waived the need for informed consent.

## Conflicts of Interest

AdaptX is the institution's preferred software tool for EHR QI data extraction and analyses. Lynn Martin is CMO and equity owner of AdaptX. No other authors have any conflicts to report.

## Supporting information


**Figure S1:** C‐Chart of the quarterly ASC case counts. Annotations highlight significant QI projects and external events that impacted this metric.
**Figure S2:** The average morphine milligram equivalent (MME). Cohorts are separated only when SCV is seen. Red dots represent single point SCV. (A) X‐bar chart and (B) S‐chart of MME in the intraoperative period. (C) X‐bar chart and (D) S‐chart of the MME in the PACU. Significant reduction in dosage and variability are seen.
**Figure S3:** Two components of our enhanced recovery program (ERP) included home administration of (A) carbohydrate containing clear fluid in this P′‐chart and (B) acetaminophen administration in this P′‐chart. Annotations highlight significant QI projects that impacted this metric. Cohorts are separated only when SCV is seen. Red dots represent single point SCV.
**Figure S4:** Prevention and treatment of postoperative nausea and vomiting was another component of the enhance recovery program (ERP). (A) P‐chart of the incidence of dual PONV prophylaxis administration for common high‐risk surgical procedures (adenotonsillectomy, arthroscopy, and strabismus repair). High reliability (> 95%) is seen with the first two procedures. (B) U‐chart representing the incidence of early PONV (defined as antiemetic treatment in the PACU) per 1000 cases. Annotations highlight significant QI projects that impacted this metric. Cohorts are separated only when SCV is seen. Red dots represent single point SCV.
**Figure S5:** Average (A) X‐bar chart and (B) S‐chart of postoperative opioid prescription dose counts for all ASC cases. Annotations highlight significant QI projects that impacted this metric. Cohorts are separated only when SCV is seen. Red dots represent single point SCV. Dose counts dropped while variability was stable.
**Figure S6:** Average (A) X‐bar chart and (B) S‐chart of postoperative opioid prescription dose counts for all adenotonsillectomy (T&A) cases only. Annotations highlight significant QI projects that impacted this metric. Cohorts are separated only when SCV is seen. Red dots represent single point SCV. Dose counts initially dropped but increase with new faculty hiring while variability was decreased with faculty retirement.
**Figure S7:** Average nitrous oxide (N_2_O) (A) X‐bar chart of volume in liters, (B) S‐chart volume in liters, and (C) P′‐chart incidence of N_2_O use. Annotations highlight significant QI projects that impacted this metric. Cohorts are separated only when SCV is seen. Red dots represent single point SCV.
**Figure S8:** U‐Chart of the rate inbound phone calls to the clinic within 14 days after surgery per 1000 cases. Annotations highlight significant QI projects and external events that impacted this metric. Cohorts are separated only when SCV is seen. Red dots represent single point SCV.
**Figure S9:** P′‐chart of the incidence of PACU normothermia (defined as temperature 36°C–38°C). Annotations highlight significant QI projects that impacted this metric. Cohorts are separated only when SCV is seen. Red dots represent single point SCV. Compliance reached the target of 90% and has been sustained for more than 8 years.

**Table S1:** Patient flow checklist content.

**Table S2:** OR safety checklists.

**Table S3:** Daily safety morning huddle.

## Data Availability

The datasets used and/or analyzed for these quality improvement initiations are available from the corresponding author upon reasonable written request.
