# Supplementary material for: Perspectives in Pediatric Ambulatory Anesthesia: Part 2—One Center's 15 Year Experience Improving Quality and Safety Outcomes
Source: Paediatr Anaesth. 2026 Jul 3;36(10):1196–206. doi: 10.1002/pan.70252 (PMC13551114; doi:10.1002/pan.70252)
Supplement: Supplementary file 1 [file PAN-36-1196-s004.pdf]

SF 1: ASC Quarterly Case Counts & Quality / External Events

n = 55,454

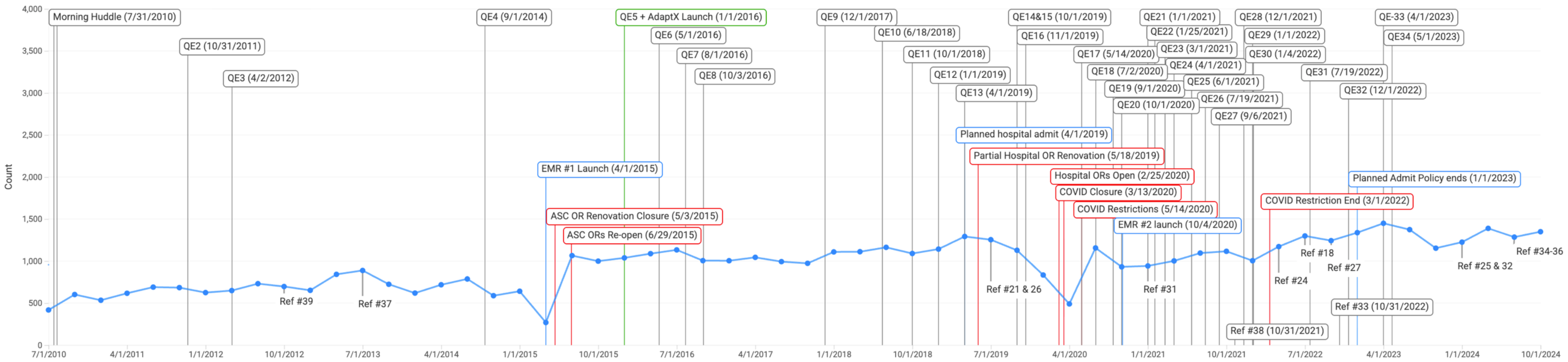

SF 2A: X-bar Chart of the Average Intraoperative Opioid Use (Morphine Milligram Equivalent)

Average MME per Kg (excluding opioid infusions); Baseline n=9,806; SCV1 n=2,787; SCV2 n=7,775

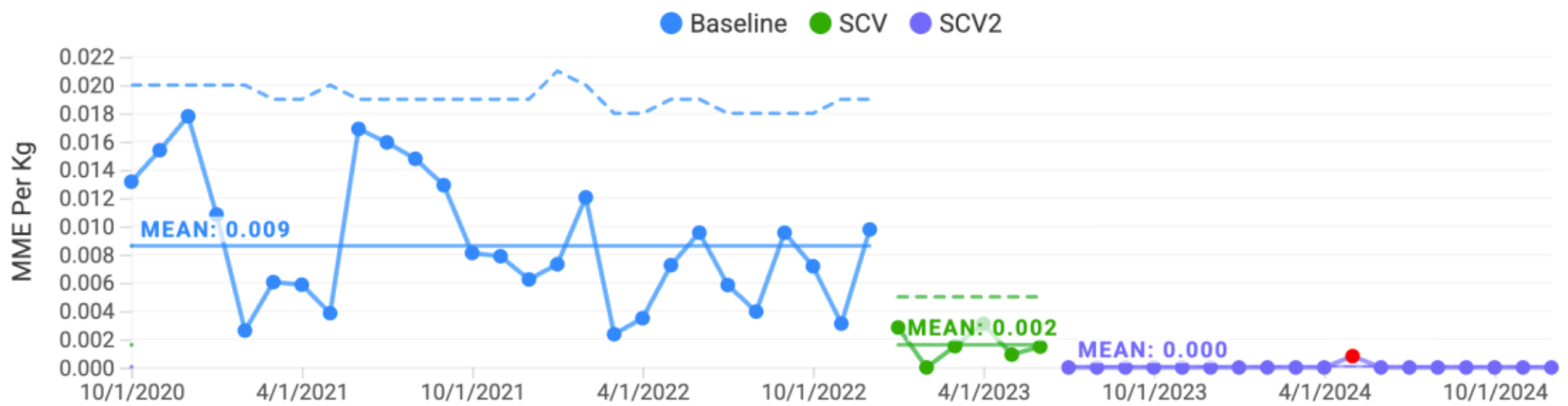

SF 2B: S-Chart of the Standard Deviation of Intraoperative Opioid Use (Morphine Milligram Equival...

Average MME per Kg (excluding opioid infusions); Baseline n=9,806; SCV1 n=2,787; SCV2 n=7,775

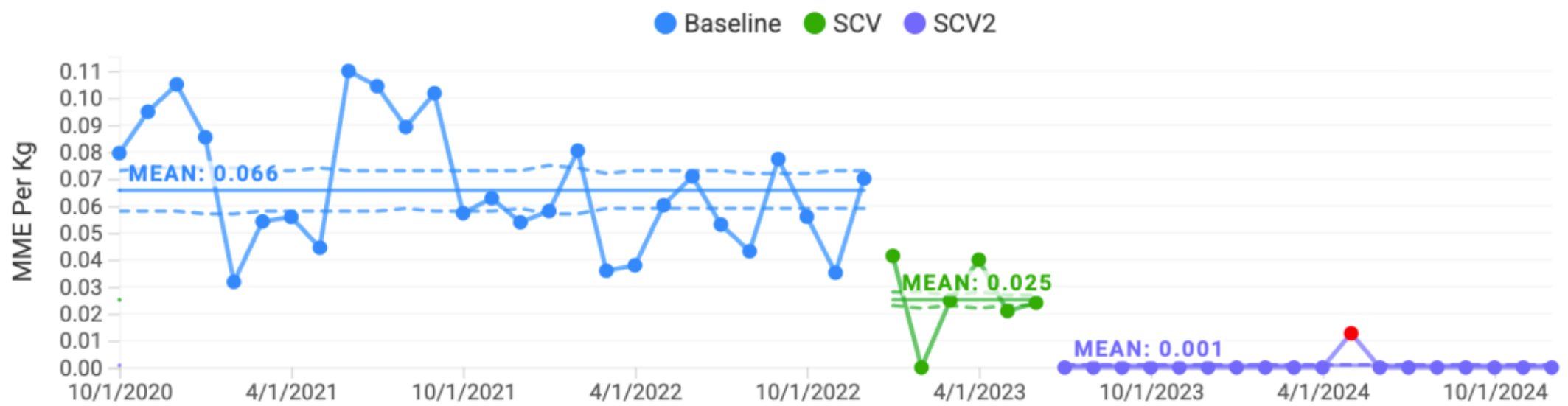

SF 2C: X-bar Chart of the Average PACU Opioid Use (Morphine Milligram Equivalent)

Average MME per Kg (excluding opioid infusions); Baseline n=6,887; SCV n=13,481

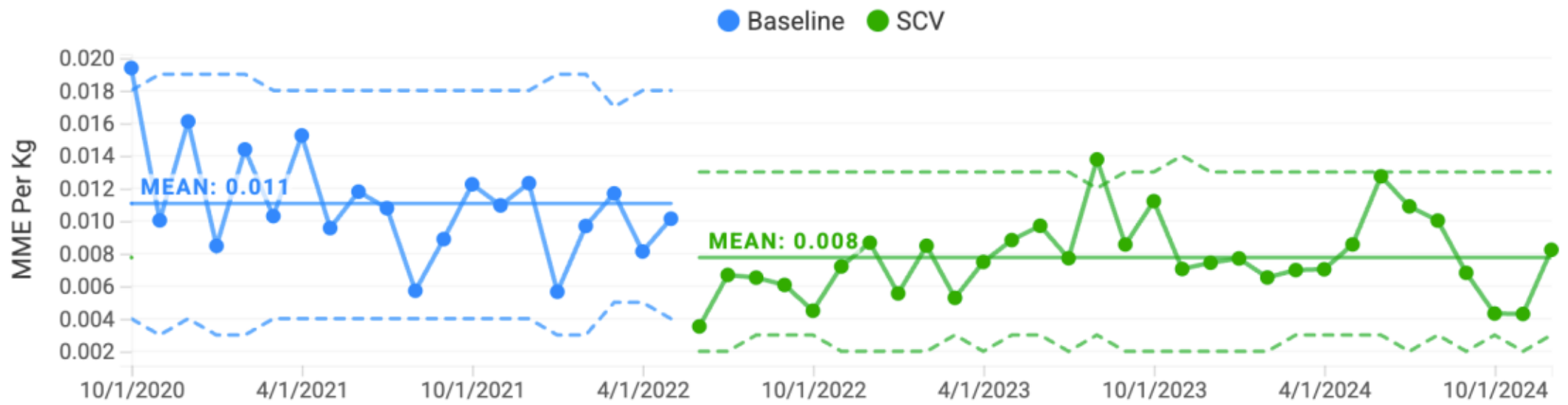

SF 2D: S-Chart of the Standard Deviation of PACU Opioid Use (Morphine Milligram Equivalent)

Average MME per Kg (excluding opioid infusions); Baseline n=6,887; SCV n=13,481

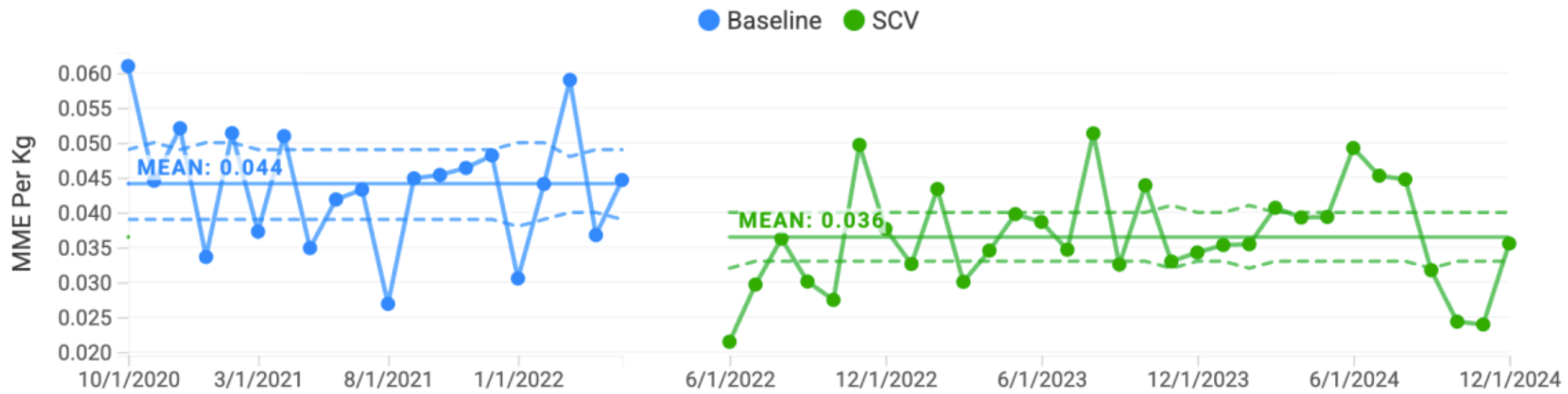

SF 3A: P Prime Chart of the Preoperative (Home) Carbohydrate Fluid Loading Rate

Y/N: Carbo juice given 2 hrs prior to surgery (Intro n=4,032; SCV1 n=12,244; SCV2 n=5,248)

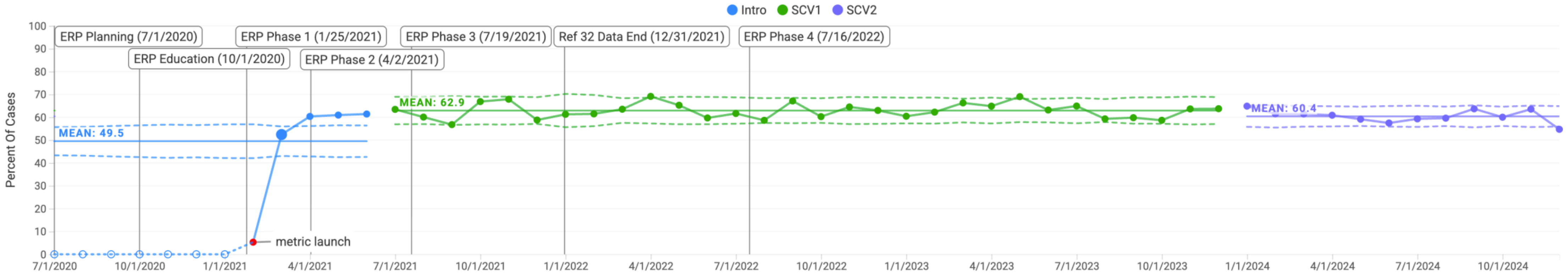

SF 3B: P Prime Chart of the Preoperative (Home) Acetaminophen Rate

n = 21,524

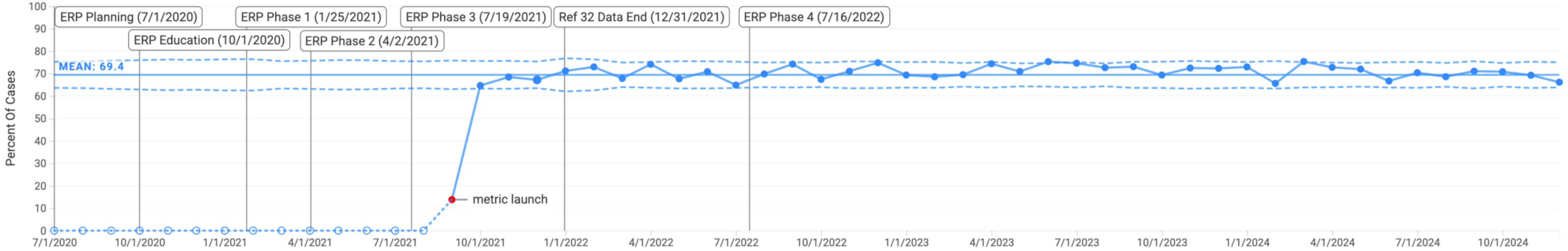

SF 4A: P-Chart of the Incidence Dual PONV Prophylaxis

Indicates administration of dual antiemetics intraop (T/A n=6,411; Arthroscopy n=1,787; Strabismus n=801) of antiemetic in PACU (T/A n=6,411; Strabismus n=801; Arthroscopy n=1,787)

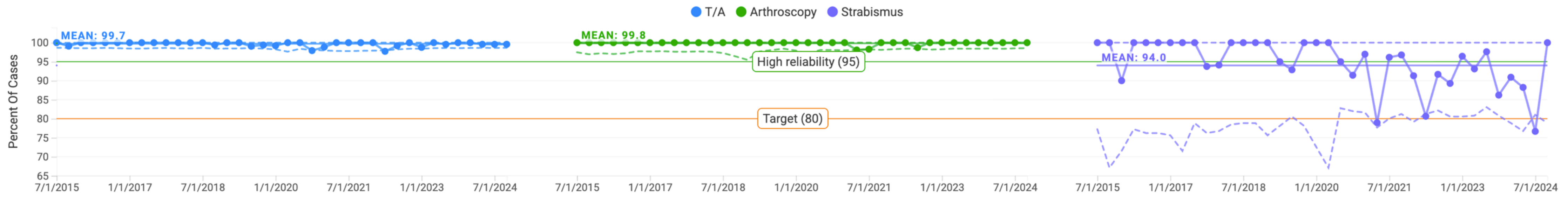

SF 4B: U-Chart of the Rate of Nausea Meds Given in PACU per 1000 Cases

Indicates administration of antiemetic in PACU (T/A n=6,411; Arthroscopy n=1,787; Strabismus n=801)

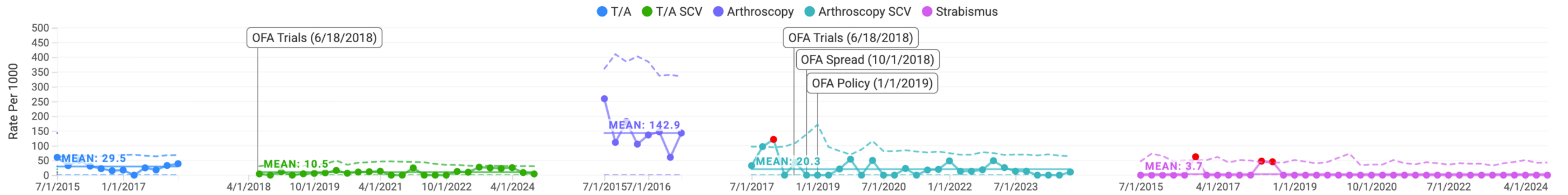

SF 5A: X-bar Chart Average Post Op Opioid Prescription Dose Count

Number of postop opioid doses prescribed by MD/dispensed by pharmacy (Baseline n=1,198; SCV1 n=724; SCV2 n=2,036)

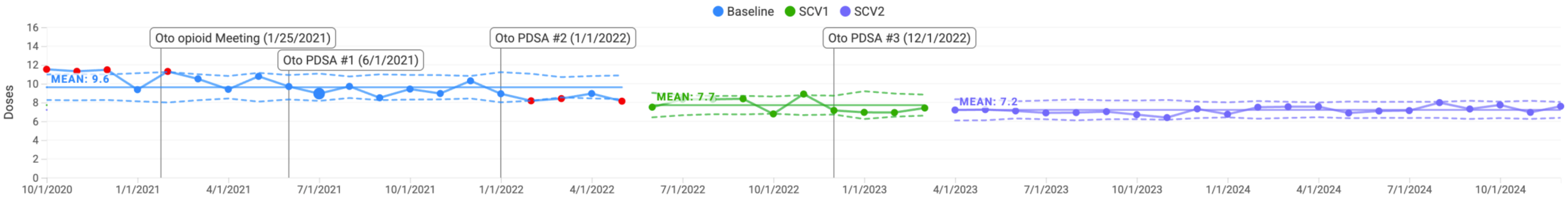

SF 5B: S-Chart of the Standard Deviation of Post Op Opioid Prescription Dose Count

Number of postop opioid doses prescribed by MD/dispensed by pharmacy (n=3.976)

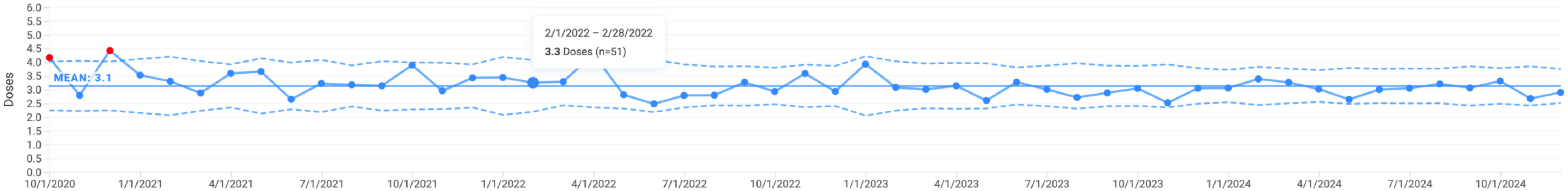

SF 6A: X-bar Chart of the Average Post Op Opioid T&A Prescription Dose Count

Number of opioid doses prescribed by MD (Baseline n=224; PDSA1 n=180; PDSA2 n=387; PDSA3 n=841; Retirement n=682; Recruitment n=236)

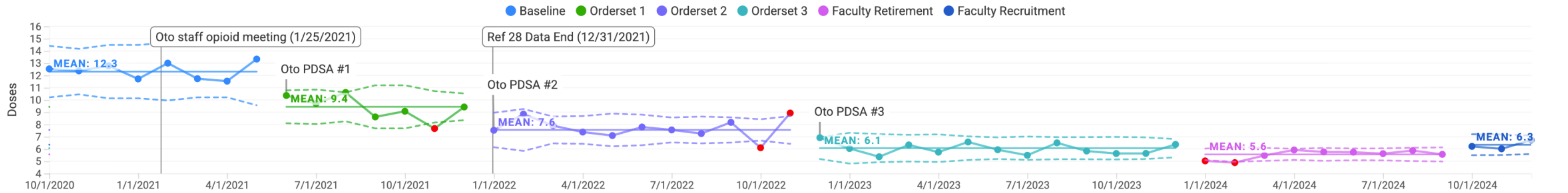

SF 6B: S-Chart of the Standard Deviation of Post Op Opioid T&A Prescription Dose Count

Number of opioid doses prescribed by MD (Baseline n=1150; SCV n=744)

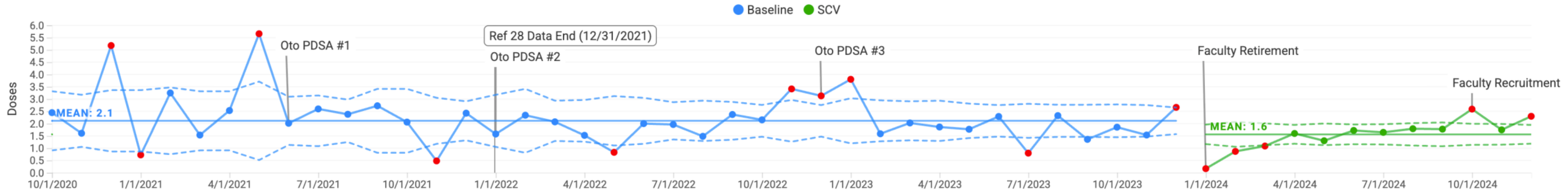

SF 7A: X-bar Chart of the Average Volume of Nitrous Oxide (liters) Used Per Case

Total liters of nitrous oxide (baseline n=4,216; SCV1 n=4,186; SCV2 n=8,873; SCV3 n=4,862; SCV4 n=1,873; SCV5 n=3,215; SCV6 n=15,280)

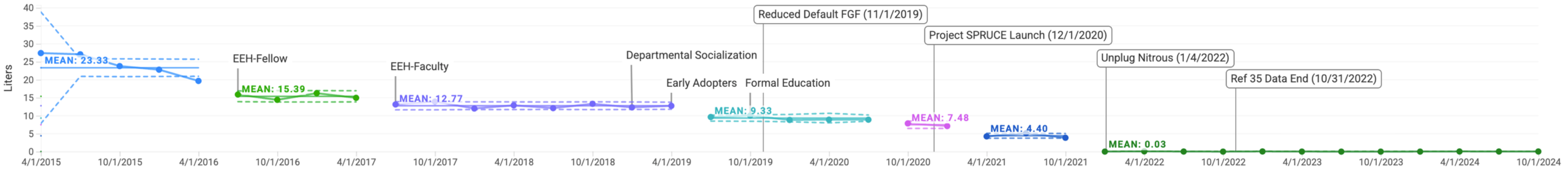

SF 7B: S-Chart of the Standard Deviation of Volume of Nitrous Oxide (liters) Used Per Case

Total liters of nitrous oxide (baseline n=1,091; SCV1 n=3,125; SCV2 n=4,186; SCV3 n=8,873; SCV4 n=5,793; SCV5 n=4,157; SCV6 n=7,505; SCV7 n=7,775)

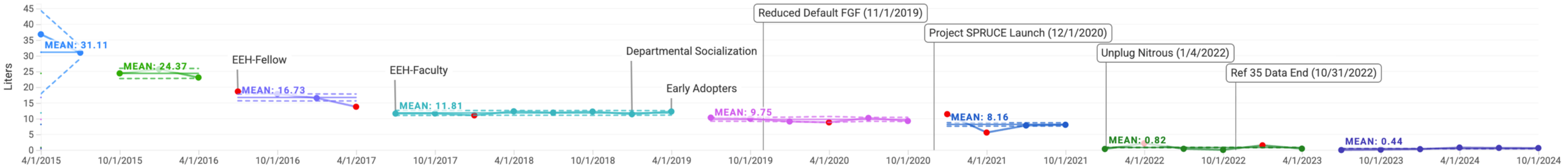

SF 7C: P Prime Chart of the Nitrous Oxide Used Rate

Nitrous oxide was used during hte procedure (Baseline n=17,275; SCV1 n=5,793; SCV2 n=4,157; SCV3 n=15,280)

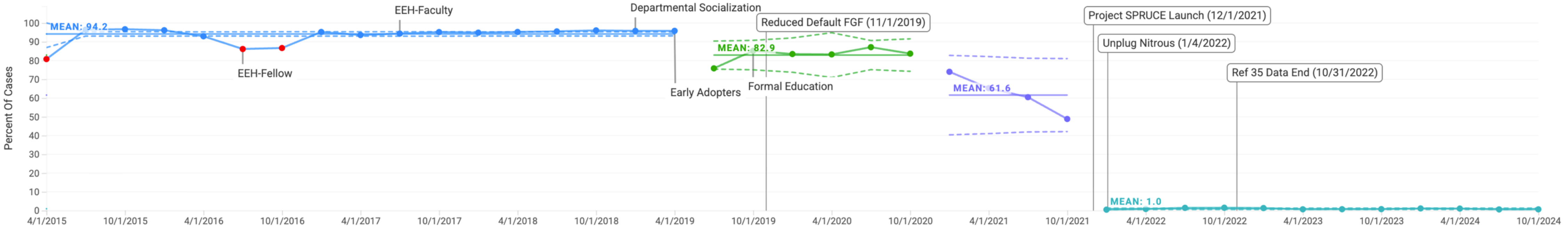

SF 8: U-Chart of the Inbound Phone Call within 14 days per 1000 Cases

Inbound call/message occurred within 14 days of surgery (Baseline n=6,887; SCV n=13,481)

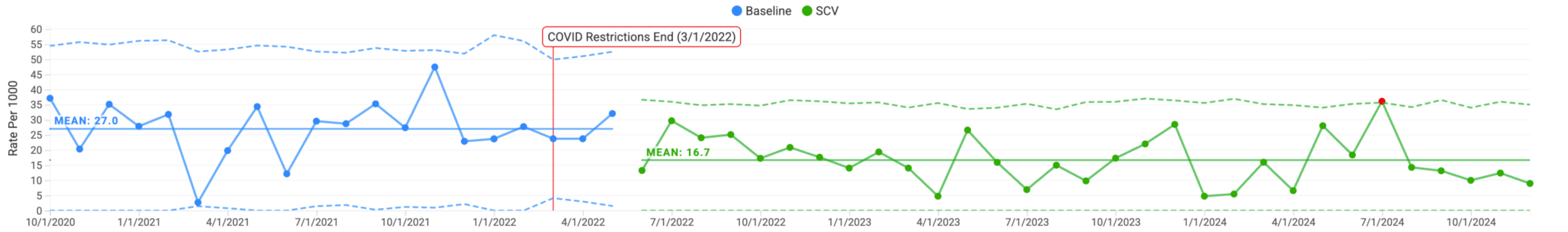

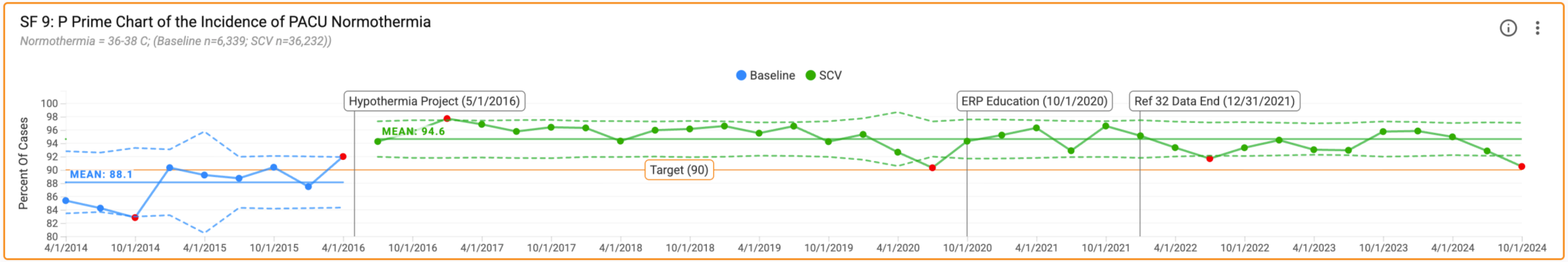
SF 9: P Prime Chart of the Incidence of PACU Normothermia
Normothermia = 36-38 C; (Baseline n=6,339; SCV n=36,232))
Baseline
SCV
Hypothermia Project (5/1/2016)
ERP Education (10/1/2020)
Ref 32 Data End (12/31/2021)
MEAN: 94.6
MEAN: 88.1
Target (90)
Percent Of Cases
100
98
96
94
92
90
88
86
84
82
80
4/1/2014
10/1/2014
4/1/2015
10/1/2015
4/1/2016
10/1/2016
4/1/2017
10/1/2017
4/1/2018
10/1/2018
4/1/2019
10/1/2019
4/1/2020
10/1/2020
4/1/2021
10/1/2021
4/1/2022
10/1/2022
4/1/2023
10/1/2023
4/1/2024
10/1/2024
