# Supplementary material for: Perspectives in Pediatric Ambulatory Anesthesia: Part 2—One Center's 15 Year Experience Improving Quality and Safety Outcomes
Source: Paediatr Anaesth. 2026 Jul 3;36(10):1196–206. doi: 10.1002/pan.70252 (PMC13551114; doi:10.1002/pan.70252)
Supplement: Supplementary file 2 [file PAN-36-1196-s001.docx]

**Supplemental Table 3: Daily Safety Morning Huddle**

**Charge RN Launch**

- Today’s daily safety huddle
- Mute your phone
- Wait for your turn in roll call
- Call out only high-risk/non-routine items only

**Frontline Leaders Today**

- OR Charge (state name)
- PACU Charge (state name)
- SPD Lead (state name)
- Anesthesia Charge (state name)
- Area leader-manager (state name)
- New team members (state name)
- Staffing call out (RED only)

**Team Roll Call:** OR1, 2, 3, 4, radiology, SPD

- Surgeon - Dr. (state name) – gives report now
- Anesthesia - Dr. (state name) – gives report now
- Nursing (state name) – gives report now
- Others?

**Improvements (Kaizen)**

- Call out any active trials
- Call out any newly approved process changes

**Announcements**

- Staffing/flow concerns
- Equipment conflicts
- Support services deficiencies
- Facilities issues
- Internal distraction events
- External events

**Summary**

- List issues still needing resolution
- Anyone have other concerns?
- Reminder to escalate problems to charge nurse
- Have a SAFE day today
