# Supplementary material for: Perspectives in Pediatric Ambulatory Anesthesia: Part 2—One Center's 15 Year Experience Improving Quality and Safety Outcomes
Source: Paediatr Anaesth. 2026 Jul 3;36(10):1196–206. doi: 10.1002/pan.70252 (PMC13551114; doi:10.1002/pan.70252)
Supplement: Supplementary file 3 [file PAN-36-1196-s002.docx]

| **Supplemental Table 2: OR Safety Checklists** | | | | | |
| --- | --- | --- | --- | --- | --- |
| (1) **SAFETY** **SIGN-IN** | | | | | |
| **WHO** | **CHALLENGE** | **RESPONSE** | | | |
|  |  | Anesthesiologist | CRNA / Fellow | | Nurse |
| RN | Ready for procedure button |  | Checked | |  |
|  | Patient ID | Matches Consent | Matches Computer | | Reads Wristband  (name, DOB, MRN) |
|  | Consent | Reads Consent  (signed, dated, timed) |  | |  |
|  | Surgical Site | All Verify Patient Marked / NA | | | |
|  | Monitor |  | Associated | |  |
|  | Room Ready |  |  | | Yes / Hold |
|  | H&P Verified |  | Yes / Verified | |  |
|  | NPO |  | Appropriate / No | |  |
|  | HCG |  | Resulted / NA | |  |
|  | Allergies |  | No / Yes -specify | |  |
|  | Difficult Airway |  | No / Yes -specify | |  |
|  | Spine at Risk |  | No / Yes -specify | |  |
|  | Regional |  | No / Yes -specify | |  |
|  | Devices |  | No / Yes -specify | |  |
|  | Safety Meds |  | In Med Tray | |  |
|  | Any Concerns | All State Concerns | | | |
|  | Visitor Devices | Put Away | | | |
|  | Epic Scan |  | Patient Scanned | |  |
|  | | | | | |
| (2) **SAFETY** **TIMEOUT** | | | | | |
| **WHO** | **CHALLENGE** | **DONE BY** | | **RESPONSE** | |
| RN | Ready for Timeout | Anyone Not Ready | | Delay Until Ready | |
|  | Introductions | All | | Name/Role | |
|  | Read Wrist Band | CRNA/Fellow  Surgeon | | Epic Story Board-Matches Computer  Looks at Consent-  Matches Consent | |
|  | Allergies | CRNA/Fellow | | None/Yes-specify | |
|  | Consent Reads | Surgeon | | Reads procedure | |
|  | Site Marking |  |  | Point to & Call  Marking on Patient | |
|  | Antibiotics |  |  | No/Yes-specify | |
|  | Antibiotics re-dosing |  |  | No/Yes-specify | |
|  | Surgical Brief |  |  | Review | |
|  | Implants |  |  | No/Yes-specify | |
|  | Imaging |  |  | No/Yes-specify | |
|  | DVT |  |  | No/Yes-specify | |
|  | Fire Safety | All | | Specify | |
|  | Counts | Surgery Tech | | No/Yes-specify | |
|  | Anesthesia Brief | CRNA/Fellow | | Review | |
|  | Warming |  |  | No/Yes-specify | |
|  | Glucose |  |  | No/Yes-specify | |
|  | Time Check | Anesthesiologist | | Green <5 minutes late  Yellow 5-20 minutes late  Red >20 minutes late | |
|  | Concerns | All Specify Any Concerns | | | |
|  | | | | | |
| (3) **SAFETY SIGN-OUT** | | | | | |
| **WHO** | **CHALLENGE** | **DONE BY** | | **RESPONSE** | |
| Surgeon | Ready for Sign-Out | Anyone | | Delay Until Ready | |
| RN | Procedure | Surgeon | | States Procedure | |
|  | Throat pack / corneal protectors |  |  | None/Out/In | |
|  | Post-op Orders |  |  | Done/Not Done | |
| Surgeon | Counts | Surgery Tech | | Correct/Incorrect/NA | |
|  | Specimens | RN | | Labeled, Sent, instructions / NA | |
|  | Family |  |  | Paged, Location | |
|  | Anesthesia Concerns | CRNA/Fellow | | No/Yes-specify | |
|  | EBL |  |  | Agree on EBL | |
|  | Devices |  |  | No/Yes-specify | |
|  | Pain Management |  |  | Routine/Regional/Other | |
|  | Equipment Issues | All | | No / Yes-specify | |
|  | Improvements | All | | No / Yes-specify | |
|  | Thank you |  | |  | |
| RN | See Next Patient | Surgeon | | Noted | |
