# Supplementary material for: Perspectives in Pediatric Ambulatory Anesthesia: Part 2—One Center's 15 Year Experience Improving Quality and Safety Outcomes
Source: Paediatr Anaesth. 2026 Jul 3;36(10):1196–206. doi: 10.1002/pan.70252 (PMC13551114; doi:10.1002/pan.70252)
Supplement: Supplementary file 4 [file PAN-36-1196-s003.docx]

| **Supplemental Table 1: Patient Flow Checklist Content** | |
| --- | --- |
| (1) **Induction Room Departure Checklist** | |
| Epic | Record Closed |
| Medications | On bed |
| Monitor | On bed |
| Airway | On bed |
| Oxygen | ‘xxx’ PSI |
| Transport circuit | Available |
| Chart/Code sheet | On bed |
| Side rails | Up |
| Gases | Off |
| Bag | On bed |
| Filter | On bed |
| *Version 6.3, modified 3/21/22* | |
|  | |
| (2) **Operating Room Arrival Checklist** | |
| Oxygen | On at ‘xx’liters/minute |
| Anesthesia | ‘Agent’ at ‘xx’ percent |
| Monitor | Up and cycling |
| Airway | On bed/machine |
| Medications | On bed/machine |
| Suction | Present |
| OR Security | Light on |
| Epic | Patient scanned |
| *Version 6.3, modified 3/21/22* | |
|  | |
| (3) **Operating Room Departure Checklist** | |
| Epic | Record closed |
| Medications | On bed |
| Monitor | On bed |
| Airway | On bed |
| Oxygen | On or circuit available |
| Donut | Off the bed |
| Safety | Straps ON & rails UP |
| Gases | Off |
| *Version 6.3, modified 3/21/22* | |
|  | |
| (4) **Recovery Room Arrival Checklist** | |
| Pt I.D. | Bracelet confirmation |
| Oxygen | On or off/circuit available |
| Monitor | Up and cycling |
| Supplies | On table |
| *Version 6.3, modified 3/21/22* | |
|  | |
| (5) **Recovery Room Departure Checklist** | |
| Medication | Yes |
| Airway | Yes |
| Manifold | Off |
| Orders | Confirmed |
| *Version 6.3, modified 3/21/22* | |
